# Temporal variations in the distribution of self-harm episodes and methods across the Australian asylum seeker population: An observational study

**DOI:** 10.1371/journal.pmed.1003235

**Published:** 2020-08-06

**Authors:** Kyli Hedrick, Gregory Armstrong, Guy Coffey, Rohan Borschmann

**Affiliations:** 1 Centre for Mental Health, Melbourne School of Population and Global Health, Faculty of Medicine, Dentistry and Health Sciences, The University of Melbourne, Carlton, Victoria, Australia; 2 Community-Minded Psychological Services, Kingsville, Melbourne, Victoria, Australia; 3 Nossal Institute for Global Health, Melbourne School of Population and Global Health, The University of Melbourne, Melbourne, Australia; 4 The Victorian Foundation for Survivors of Torture (Foundation House), Brunswick, Victoria, Australia; 5 Justice Health Unit, Centre for Health Equity, Melbourne School of Population and Global Health, Faculty of Medicine, Dentistry and Health Sciences, The University of Melbourne, Carlton, Victoria, Australia; 6 Centre for Adolescent Health, Murdoch Children’s Research Institute, Parkville, Victoria, Melbourne, Australia; 7 Health Service and Population Research Department, Institute of Psychiatry, Psychology and Neuroscience, King’s College London, London, United Kingdom; 8 Melbourne School of Psychological Sciences, The University of Melbourne, Melbourne, Australia; International Organization for Migration, SRI LANKA

## Abstract

**Background:**

Temporal patterns in the frequency and characteristics of self-harm episodes across the Australian asylum seeker population may have implications for self-harm prevention and public health policy. The aim of this study was to examine how the distribution of self-harm episodes and method(s) of self-harm used across the Australian asylum seeker population vary according to the 24-hour cycle, day, and month, and to establish a basis for further research.

**Methods and findings:**

We conducted an observational study of all 949 self-harm incidents reported across the Australian asylum seeker population (representing a monthly average of 28,992 adults) between 1 August 2014 and 31 July 2015, obtained by Freedom of Information (FOI) from the Department of Immigration. Time of self-harm, day, and month of occurrence were investigated across all five Australian asylum seeker populations (i.e., community-based arrangements, community detention, onshore immigration detention, offshore immigration detention [Nauru], and offshore immigration detention [Manus Island]). Significant variations in distributions over the 24-hour cycle were observed by processing arrangements. Compared with the average distribution across all other processing arrangements, self-harm more commonly occurred among community-based asylum seekers (36.3%) between 12:00 AM and 3:59 AM (*p* < 0.001), in asylum seekers on Manus Island (36.4%) between 4:00 PM and 7:59 PM (*p* = 0.02), and among asylum seekers in onshore detention (20.4%) between 8:00 PM and 11:59 PM (*p* < 0.001). Compared with the average distribution across all other methods, self-poisoning (by medication) (25%) was significantly more likely to occur between 12:00 AM and 3:59 AM (*p* = 0.009), and self-battery (42%) between 8:00 AM and 11:59 AM (*p* < 0.001). The highest and lowest monthly self-harm episode rates for the whole asylum seeker population were in August (2014) (5 episodes per 1,000 asylum seekers; 95% confidence interval [CI] 1–11) and in both January and February (2015) (2.1 episodes per 1,000 asylum seekers; 95% CI 0.6–7.2), respectively; however, the overlapping CIs indicate no statistically significant differences across the months. When examining monthly trends by processing arrangements, we observed that self-harm was significantly more likely to occur in August (2014) than other months of the year among asylum seekers in onshore detention (19%) (*p* < 0.001), in January (2015) on Manus Island (18%) (*p* = 0.002), and in October (2014) on Nauru (15%) (*p* < 0.001). The main study limitations were that we could not investigate certain characteristics associated with self-harm (e.g., gender, country of origin), as the Department of Immigration did not routinely collect such data. There was also the potential risk of making a type 1 error, given the exploratory nature of the comparisons we undertook; we minimised this by lowering our significance threshold from 0.05 to 0.01.

**Conclusions:**

Self-harm in the Australian asylum seeker population was found to vary according to time of day and month of the year, by processing arrangements. A series of procedure-related and detention-related factors were observed to be associated with the temporal variations in self-harm. These findings should form the basis for further investigation into temporal variations in self-harm among asylum seekers, which may in turn lead to effective self-harm prevention strategies.

## Abstract 2

In an observational study, Kyli Hedrick and colleagues investigate hourly, daily, and monthly patterns in reported self-harm incidents across the Australian asylum seeker population.

## Author summary

**Why was this study done?:**

- Research indicates that the mental health of asylum seekers is affected by stressors experienced before, during, and after the migration process; however, until recently, little has been known about self-harm among asylum seekers, due to the lack of accessible data.
- Research in 2019 has shown that rates of self-harm among people seeking asylum in Australia, including those on the Pacific island nation of Nauru and Manus Island (Papua New Guinea), are exceptionally high compared with the general Australian community.
- This research tells us about the extent of self-harm in asylum seekers, but it does not tell us anything about the timing of these self-harm episodes. Information about when self-harm occurs might highlight particular stressors, including those related to policies, practices, or the environment, which could then be targeted to reduce self-harm in future.

**What did the researchers do and find?:**

- We examined all self-harm incidents that were reported as occurring in the Australian asylum seeker population from 1 August 2014 to 31 July 2015, obtained via Freedom of Information (*N* = 949). Time of day, day of the week, and month of the year were investigated in all 949 incidents, according to the five detained and community-based asylum seeker populations in Australia (including Nauru and Manus Island).
- Significant variations in the distribution of self-harm over the 24-hour cycle were observed across some asylum seeker populations.
- While for the entire asylum seeker population there were no significant variations in rates of self-harm by month of the year, self-harm was significantly more likely to occur in August 2014 (than other months of the year) among asylum seekers in onshore detention (19%), in January 2015 on Manus Island (18%), and in October 2014 on Nauru (15%). Compared with all other methods, self-poisoning (by medication) (25%) was significantly more likely to occur between 12:00 AM and 3:59 AM, and self-battery (42%) between 8:00 AM and 11:59 AM.

**What do these findings mean?:**

- In this study, we observed that self-harm varied according to time of day and month of the year, including by the different asylum seeker populations.
- Future research should examine these temporal trends in further detail, in order to inform self-harm prevention strategies, including those at the policy level.
- Ongoing self-harm monitoring practices should be established to more closely track and identify temporal (and other) trends, in order to protect asylum seekers from further harm.

## Introduction

Global and regional conflicts have resulted in an increase in the numbers of forcibly displaced people worldwide [1]. It is now established practice for many countries to detain asylum seekers whilst their claims for protection are processed [2]. Australia has had a policy of mandatory immigration detention for all ‘unlawful non-citizens’, which includes those seeking asylum, for over 25 years [3]. Since August 2012, asylum seekers who arrived by boat have been subject to offshore processing on the Pacific island nation of Nauru and Manus Island, Papua New Guinea (PNG) [4]. From late 2012 onwards, depending on mode and date of arrival, there have been five main Australian asylum seeker populations, categorised according to processing arrangements: (a) community-based asylum seekers; (b) those who are held in community detention; (c) those held in onshore immigration detention (which includes centres on the Australian mainland as well as on Christmas Island, a remote island located in the Indian Ocean); (d) those who are held in offshore immigration detention on Nauru; and (e) those held in offshore immigration detention on Manus Island [5].

Offshore processing on Nauru and Manus Island has been the subject of sustained commentary and criticism in recent years [4]. This has been mainly due to the remote location of both islands, poor detention conditions, the indefinite length of detention for asylum seekers held there, lack of access to adequate legal assistance, and limited resettlement options for asylum seekers and refugees [4]. The standard and accessibility of mental and primary healthcare services in detention centres have also been queried by health and human rights organisations [4,6]. Physical and sexual violence against asylum seekers and refugees on Nauru and Manus has also been reported [7]. A number of similar concerns regarding conditions in onshore immigration detention on the Australian mainland (including Christmas Island) have also been made by a range of allied health professionals, academics, as well as human rights organisations and other relevant health bodies [8]. Conditions in community detention (where asylum seekers—generally vulnerable adults, and families—are required to reside in a specified location, with strict supervision requirements) and for community-based asylum seekers (who may live in a place of their own choosing) are likely to mitigate some of the harms associated with closed immigration detention. Concerns regarding the impact of restrictions associated with both of these types of community-based processing arrangements, however, such as limitations on study and work rights, no or intermittent access to healthcare, access to legal support, little or no income support, food insecurity, as well as insecurity of housing for some community-based asylum seekers, have also been frequently raised [9].

Research has consistently found that asylum seekers are vulnerable to poor mental health, including higher rates of post-traumatic stress disorder (PTSD), anxiety, and depression, compared with the general community [10]. This is due, in part, to premigration stressors, including torture and trauma. Post-migratory stress is also an important predictor of mental health problems, and in some cases, even more so than pre-migratory stress. Such stressors include traumatic flight and detention experiences, prolonged separation from family, and visa, housing, food, and employment insecurity [10]. Until recently, however, knowledge about the epidemiology of self-harm among asylum seekers has been limited, mainly as a consequence of the lack of accessible data [5].

Following the release of the largest set of de-identified self-harm data from across the entire Australian asylum seeker population via Freedom of Information (FOI)—and the first since offshore processing recommenced in late 2012—we recently investigated the episode rates and characteristics of self-harm across the entire Australian asylum seeker population between 1 August 2014 and 31 July 2015 [5]. This research—involving the same cohort and time frame of relevance to the present study—highlighted the extraordinarily high rates of self-harm among detained asylum seekers in comparison with rates found among the general Australian community, and in asylum seekers in community-based settings [5]. Calculated annual episode rates of self-harm among asylum seekers in Manus Island (54 per 1,000 detained asylum seekers), onshore detention (257 per 1,000 detained asylum seekers), and Nauru (260 per 1,000 detained asylum seekers) were 45 times, 214 times, and 216 times the Australian hospital-treated rates for self-harm, respectively [5]. By comparison, episode rates among asylum seekers in community-based arrangements (5 per 1,000) and community detention (27 per 1,000) for this one-year period were 4 and 22 times the Australian community rates for hospital-treated self-harm, respectively [5]. As we strongly asserted [5], these findings highlight the deleterious effects of immigration detention on the health of asylum seekers and warrant urgent attention.

Whilst our earlier findings demonstrate that asylum seekers, particularly those in held or ‘closed’ detention, are at a higher risk of self-harm than asylum seekers in community-based settings, they do not provide us with information about the timing of these self-harm episodes or thus any details about the potential association with policies, conditions, or practices that increase asylum seekers’ vulnerability. As knowledge of this type may guide the development of self-harm prevention strategies, including at the national policy level, the timing of self-harm episodes would seem important to explore. According to self-harm reporting guidelines from the World Health Organization (WHO) [11]—outlined in a detailed practice manual—there are a number of core self-harm data items that should be routinely collected and reported on by countries ‘to guide and prioritise the best interventions in each context and contribute to an effective… self-harm prevention strategy’ (p. 3). Prevention strategies include those related to public policy [11]. Three of the core WHO data items of relevance to the Australian asylum seeker population focus on the temporal dimensions of self-harm: time of self-harm, day of the week, and date (including month of the year) [11]. As we have argued elsewhere [12], the independent monitoring of self-harm among asylum seekers in Australia, in accordance with WHO self-harm reporting guidelines, is urgently needed to highlight trends and develop prevention strategies, but is not being conducted. The aim of this exploratory study, consequently, was to examine how the distribution of self-harm episodes, and method(s) of self-harm used across the Australian asylum seeker population, vary according to the 24-hour cycle, day of the week, and month of the year, including by processing arrangements, and to establish a basis for further research and self-harm monitoring practices, which in turn may be used to inform future self-harm prevention strategies.

## Methods

This study is reported as per the Strengthening the Reporting of Observational Studies in Epidemiology (STROBE) guideline (S1 STROBE Checklist).

All self-harm incidents reported in the Australian asylum seeker population between 1 August 2014 and 31 July 2015 were obtained under the *Freedom of Information Act* (Commonwealth of Australia) [13], after being de-identified and published on the FOI disclosure log of the (then-called) Department of Immigration and Border Protection (DIBP) [14]. Ethics approval for this study was granted by the University of Melbourne’s Human Research Ethics Committee (#1749949.1).

Self-harm is defined as all forms of intentional self-injury (or self-poisoning), irrespective of suicidal intent or motivation [15,5]. Based on the contractual obligations the DIBP has with detention centre and community-based staff or contractors, all episodes of self-harm in the Australian asylum seeker population are required to be reported verbally to a duty manager within 30 minutes, and in writing on an incident report form within three hours [15]. The incident reports are then entered into the DIBP’s incident management portal, where they are archived. Internal guidelines released on the DIBP’s FOI disclosure log [14] indicate that the reports are supposed to contain a description of the self-harm incident, the nature of the injury and method used, and the incident’s time and place, as well as any action taken. As highlighted elsewhere [5], it is possible that self-harm incidents in the Australian asylum seeker population are underreported due to data management, supervision, and recording practices. Furthermore, as most self-harm does not lead to medical-help seeking, it is almost certainly underreported in this context [5]. Instances of suicide were not included in the analysis, as deaths are not recorded in this category of incident reports. The most definitive record of deaths amongst Australian asylum seekers—the Australian Border Deaths Observatory [16]—indicates there were six deaths between 1 August 2014 and 31 July 2015, of which three were recorded as suicides.

The methodological approach used in the present study was content analysis. Parts of this methodology have been described elsewhere [5]. Each of the self-harm incident reports were categorised according to processing arrangements (that is, community-based arrangements, community detention, onshore detention, offshore detention [Nauru], and offshore detention [Manus Island]), method(s) used to self-harm, time of self-harm episode, day of the week, month, and gender. Time, date, and month of self-harm episode were obtained from the information routinely entered on each incident report, and day of the week was extrapolated from this information. Given that the self-harm incident reports did not contain a gender tick box, gender was coded following a qualitative analysis of the text in each report. Cases for which gender was not able to be established were classified as a distinct category of ‘gender not known’. As a reliability check, an independent coder was used to assess the way a subsample of 100 incident reports were categorised. The inter-rater reliability was found to be very high (kappa = 0.95) [17] and, on that basis, all remaining events were coded by a single coder only. Table 1 outlines the estimated average adult population figures for the 12-month period by processing arrangements and gender, according to the DIBP’s [18] statistics.

**Table 1:** Monthly average number of adults in the Australian asylum seeker population between 1 August 2014 and 31 July 2015, by processing arrangements and gender.

Processing arrangements	Male population	Female population	Total population**
Community-based	21,136	2,758	23,894
Community detention	622	569	1,191
Onshore detention	1,815	361	2,176
Nauru	564	159	723
Manus Island*	1,008	-	1,008

*Manus Island houses only male asylum seekers.

**The total average population figures reported here vary slightly (by <0.03%) from those we have reported elsewhere [5] due to a lag in the DIBP’s monthly versus annual reporting processes, including across locations.

Abbreviation: DIBP, Department of Immigration and Border Protection

Data were analysed using SPSS version 24 (IBM, Armonk, NY). Chi-squared tests were used to establish whether the observed differences between characteristics and temporal patterns were statistically significant. In order to reduce the potential risk of type 1 errors, given the number of exploratory comparisons undertaken, we lowered our significance threshold from 0.05 to 0.01 [19]. Monthly episode rates of self-harm per 1,000 asylum seekers were calculated for the total asylum seeker population, as well as for three of the five main populations, according to the DIBP’s [18] statistics, with 95% confidence intervals (CI) based on Poisson distribution. Numbers were too low to calculate monthly episode rates for community detention. The monthly episode rates of self-harm (with 95% CIs) are displayed in S1 Table and S2 Table. Time of day was divided into six 4-hour intervals: 12:00 AM–3:59 AM, 4:00 AM–7:59 AM, 8:00 AM–11:59 AM, 12:00 PM–3:59 PM, 4:00 PM–7:59 PM, and 8:00 PM–11:59 PM.

No prospective analytic protocol was created. The main research question regarding the temporal variations in self-harm by the 24-hour cycle, day, and month of the year—and self-harm methods used across these temporal dimensions—was formulated prior to data inspection and from the literature highlighted in the introduction. Subsequent analyses were also planned to disaggregate the analyses by processing arrangements, given our previous findings [5] that the self-harm episode rates varied substantially across the different settings. Initial inspections of our statistically significant results showed that all except one had *p*-values of <0.001. Given the number of comparisons made, however, there was the potential risk of making a type 1 error. In response to reviewer feedback about this, we subsequently lowered our significance threshold from 0.05 to 0.01. We also supplemented the core results with monthly episode rates of self-harm in order to support the variations in distributions found, based on reviewer comments.

## Results

### Study sample

During the 12-month period from 1 August 2014 to 31 July 2015, a total of 949 episodes of self-harm were recorded as occurring across the entire Australian asylum seeker population. Of the 949 episodes of self-harm, 560 episodes (59.0%) involved the 2,176 asylum seekers detained in onshore immigration detention, 188 episodes (19.8%) involved the 723 asylum seekers held on Nauru, 113 episodes (11.9%) involved the 23,894 asylum seekers in community-based arrangements, 55 episodes (5.8%) involved the 1,008 asylum seekers held on Manus Island, and 33 episodes (3.5%) involved the 1,191 asylum seekers held in community detention. Details regarding gender were able to be extracted from 590 (62.1%) of the 949 self-harm incident reports. Gender was not able to be determined in 359 (37.9%) episodes. Males were involved in 426 (72.2%) self-harm episodes (where gender was able to be determined) and females in 164 (27.8%). No children or adolescents were included in the study sample, as data pertaining to minors were not available. Information regarding country of origin, age, date of birth, length of detention, psychiatric history, or suicidal intent was not able to be extracted from the self-harm incident reports.

### The proportion of self-harm episodes by 24-hour cycle

The time of occurrence was recorded for all 949 self-harm episodes (Table 2). Self-harm was found to vary according to time of day across the Australian asylum seeker population (χ^2^ [20, *N* = 949] = 107.2, *p* < 0.001). On average, across all processing arrangements self-harm most commonly occurred across two consecutive four-hour intervals—between 12:00 PM and 7:59 PM—during which time 46.2% of all self-harm episodes (*n* = 438) occurred. The peak time was between 12:00 PM and 3:59 PM, when 25.3% of all self-harm episodes (*n* = 240) occurred. The trough in self-harm episodes occurred between 4:00 AM and 7:59 AM, during which time 6.8% of all self-harm episodes (*n* = 65) occurred. Following this time, self-harm episodes increased in frequency until the late afternoon peak time.

**Table 2:** The number and percentage of self-harm episodes in the Australian asylum seeker population between 1 August 2014 and 31 July 2015, by processing arrangements, according to time of day, day of the week, and month of occurrence.

Number of self-harm episodes	Onshore detention	Nauru	Manus Island	Community detention	Community-based	*N* (%)
	*n* (%)	*n* (%)	*n* (%)	*n* (%)	*n* (%)	
	560	188	55	33	113	949
**Time of day**						
12:00 AM–3:59 AM	49 (8.8)	25 (13.3)	6 (10.9)	5 (15.2)	41 (36.3)	126 (13.3)
4:00 AM–7:59 AM	47 (8.4)	7 (3.7)	1 (1.8)	1 (3.0)	9 (8.0)	65 (6.8)
8:00 AM–11:59 AM	93 (16.6)	28 (14.9)	6 (10.9)	6 (18.2)	19 (16.8)	152 (16.0)
12:00 PM–3:59 PM	141 (25.2)	48 (25.5)	9 (16.4)	7 (21.2)	35 (31.0)	240 (25.3)
4:00 PM–7:59 PM	116 (20.7)	49 (26.1)	20 (36.4)	7 (21.2)	6 (5.3)	198 (20.9)
8:00 PM–11:59 PM	114 (20.4)	31 (16.5)	13 (23.6)	7 (21.2)	3 (2.7)	168 (17.7)
**Day**						
Monday	71 (12.7)	20 (10.6)	7 (12.7)	3 (9.1)	18 (15.9)	119 (12.5)
Tuesday	86 (15.4)	28 (14.9)	7 (12.7)	8 (24.2)	20 (17.7)	149 (15.7)
Wednesday	80 (14.3)	30 (16.0)	13 (23.6)	4 (12.1)	17 (15.0)	144 (15.2)
Thursday	105 (18.8)	24 (12.8)	11 (20.0)	6 (18.2)	15 (13.3)	161 (17.0)
Friday	77 (13.8)	36 (19.1)	6 (10.9)	6 (18.2)	14 (12.4)	139 (14.6)
Saturday	76 (13.6)	26 (13.8)	6 (10.9)	1 (3.0)	13 (11.5)	122 (12.9)
Sunday	65 (11.6)	24 (12.8)	5 (9.1)	5 (15.2)	16 (14.2)	115 (12.1)
**Month**						
August 2014	109 (19.5)	12 (6.4)	4 (7.3)	7 (21.2)	16 (14.2)	148 (15.6)
September 2014	57 (10.2)	19 (10.1)	1 (1.8)	3 (9.1)	13 (11.5)	93 (9.8)
October 2014	30 (5.4)	29 (15.4)	1 (1.8)	2 (6.1)	17 (15.0)	79 (8.3)
November 2014	41 (7.3)	12 (6.4)	7 (12.7)	-	9 (8.0)	69 (7.3)
December 2014	40 (7.1)	12 (6.4)	6 (10.9)	1 (3.0)	10 (8.8)	69 (7.3)
January 2015	25 (4.5)	17 (9.0)	10 (18.2)	3 (9.1)	7 (6.2)	62 (6.5)
February 2015	37 (6.6)	15 (8.0)	3 (5.5)	2 (6.1)	5 (4.4)	62 (6.5)
March 2015	33 (5.9)	17 (9.0)	6 (10.9)	4 (12.1)	5 (4.4)	65 (6.8)
April 2015	48 (8.6)	15 (8.0)	2 (3.6)	2 (6.1)	3 (2.7)	70 (7.4)
May 2015	39 (7.0)	11 (5.9)	7 (12.7)	3 (9.1)	9 (8.0)	69 (7.3)
June 2015	55 (9.9)	13 (6.9)	4 (7.3)	3 (9.1)	11 (9.7)	86 (9.1)
July 2015	46 (8.2)	16 (8.5)	4 (7.3)	3 (9.1)	8 (7.1)	77 (8.1)

### The proportion of self-harm episodes by 24-hour cycle, according to processing arrangements

**Fig 1:**
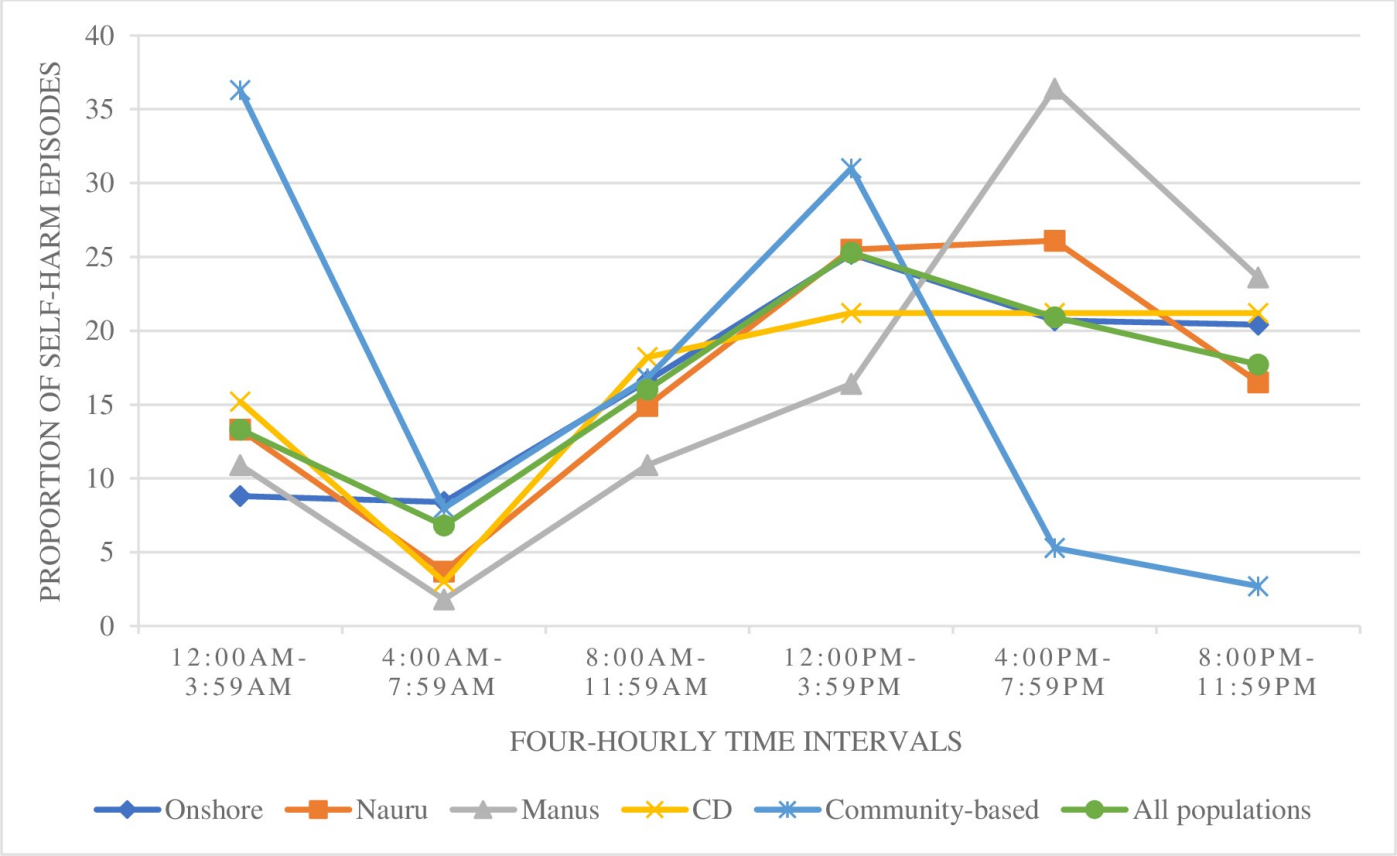
The proportion of self-harm episodes occurring across the Australian asylum seeker population per four-hourly intervals over the 24-hour time frame, by processing arrangements. Self-harm episodes occurring in asylum seekers in onshore detention, Nauru, Manus Island, community detention, community-based asylum seekers, as well as across the total Australian asylum seeker population, according to four-hourly intervals over the 24-hour time frame. CD, community detention

The four-hourly pattern of self-harm episodes was also examined according to processing arrangements. The distributions across the 24-hour cycle (by four-hourly intervals) varied markedly by processing arrangements (Fig 1). The pattern for community-based asylum seekers was such that self-harm more commonly occurred between 12:00 AM and 3:59 AM (36.3%), compared with the average distribution across all other processing arrangements (10.2%) (χ^2^ [5, *N* = 949] = 84.3, *p* < 0.001). Self-harm episodes among asylum seekers in Manus Island more commonly occurred between 4:00 PM and 7:59 PM (36.4%), compared with the average distribution across all other processing arrangements (19.9%) (χ^2^ [5, *N* = 949] = 13.0, *p* = 0.02); however, this difference did not reach statistical significance. The pattern of self-harm episodes for asylum seekers in onshore immigration detention was such that self-harm more commonly occurred between 8:00 PM and 11:59 PM (20.4%), compared with the average distribution across all other processing arrangements (13.9%) (χ^2^ [5, *N* = 949] = 31.5, *p* < 0.001). The temporal pattern of self-harm episodes for asylum seekers in Nauru (χ^2^ [5, *N* = 949] = 6.76, *p* = 0.238) or in community detention (χ^2^ [5, *N* = 949] = 1.56, *p* = 0.906), however, did not differ significantly from the average distribution across all other processing arrangements.

### Self-harm episodes by day of the week, including by processing arrangements

The date (from which day of the week was derived) was recorded for all 949 self-harm episodes. The distribution of self-harm episodes did not vary significantly according to day of the week or by processing arrangements (χ^2^ [24, *N* = 949] = 20.0, *p* = 0.693). Table 2 displays the proportion of self-harm episodes by day of the week, including by processing arrangements.

### Self-harm episodes by month of occurrence

The month of occurrence was recorded for all 949 self-harm episodes (Table 2). The monthly average number of self-harm episodes across the Australian asylum seeker population was 79 (calculated by dividing the total number of episodes by 12). Nearly double the average number of self-harm episodes were recorded in August (148), with above-average numbers also recorded in September (93) and June (86). January (62) and February (62) both recorded below-average numbers. Whilst self-harm was found to occur more commonly in August compared with the distribution across all other months (χ^2^ [4, *N* = 949] = 22, *p* < 0.001), across parts of the Australian asylum seeker population, the residuals highlight that this was only the case for asylum seekers in onshore detention and community detention. Indeed, whilst the calculated monthly episode rates per 1,000 (95% CI) for the total asylum seeker population (see S1 Table) show that the highest rate of self-harm occurred in August 2014 (5 episodes/1,000 asylum seekers [95% CI 1–11]), the overlapping CIs do provide a strong indication that differences across the months over the whole population are unlikely to be statistically significant.

### The proportion of self-harm episodes by month of occurrence, according to processing arrangements

One fifth of all self-harm episodes in community detention (21.2%) and onshore detention (19.5%) occurred in August, close to a fifth (18.2%) of all self-harm episodes in Manus Island occurred in January, and just over a tenth of all self-harm episodes amongst community-based asylum seekers (15.0%) and in Nauru (15.4%) occurred in October (Table 2). Compared with the average distribution across all other processing arrangements, asylum seekers in onshore detention were significantly more likely to self-harm in August (χ^2^ [11, *N* = 949] = 42.2, *p* < 0.001), asylum seekers in Nauru were significantly more likely to self-harm in October (χ^2^ [11, *N* = 949] = 34.1, *p* < 0.001), and those held on Manus Island were significantly more likely to self-harm in January (χ^2^ [11, *N* = 949] = 29.3, *p* = 0.002). There were no significant differences between the average distribution across all processing arrangements and month of occurrence for community-based asylum seekers (χ^2^ [11, *N* = 949] = 6.2, *p* = 0.855). Cell counts were too low to test for significant differences amongst asylum seekers in community detention by month of occurrence. Table 2 displays self-harm by month of occurrence, according to processing arrangements. Calculated monthly episode rates of self-harm per 1,000 (95% CI) (see S2 Table) by processing arrangements support the pattern of distribution observed: episode rates were highest among asylum seekers on Nauru in October (31 episodes/1,000 asylum seekers [95% CI 21–44]), on Manus Island in January (9 episodes/1,000 asylum seekers [96% CI 4–17]), and in onshore detention in August (39 episodes/1,000 asylum seekers [95% CI 27–53]).

### Methods of self-harm by 24-hour cycle

Methods of self-harm were documented in 774 (81.5%) of the 949 self-harm incident reports. The three most common methods of self-harm were as follows: cutting (290 episodes, 37.4%), self-battery (199 episodes, 26.0%), and attempted hanging (83 episodes, 11.0%). Table 3 outlines the descriptive statistics for all methods of self-harm reported as being used among the Australian asylum seeker population during the 12-month period.

**Table 3:** Methods of self-harm used by the Australian asylum seeker population between 1 August 2014 and 31 July 2015, according to time of day, day of the week, and month of occurrence.

Methods of self-harm	Cutting	Self-battery	Attempt. hanging	Self-poisoning (medication)	Self-poisoning (chemical)	Ingest foreign object	Other	*N* (%)
	*n* (%)	*n* (%)	*n* (%)	*n* (%)	*n* (%)	*n* (%)	*n* (%)	
	290	199	83	79	57	28	38	774
**Time of day**								
12:00 AM–3:59 AM	38 (13.1)	9 (4.5)	15 (18.1)	20 (25.3)	7 (12.3)	3 (10.7)	4 (10.5)	96 (12.4)
4:00 AM–7:59 AM	16 (5.5)	15 (7.5)	3 (3.6)	4 (5.1)	5 (8.8)	1 (3.6)	3 (7.9)	47 (6.1)
8:00 AM–11:59 AM	50 (17.2)	42 (21.1)	9 (10.8)	8 (10.1)	8 (14.0)	4 (14.3)	6 (15.8)	127 (16.4)
12:00 PM–3:59 PM	83 (28.6)	49 (24.6)	19 (22.9)	16 (20.3)	13 (22.8)	7 (25.0)	6 (15.8)	193 (24.9)
4:00 PM–7:59 PM	57 (19.7)	40 (20.1)	22 (26.5)	15 (19.0)	15 (26.3)	10 (35.7)	12 (31.6)	171 (22.1)
8:00 PM–11:59 PM	46 (15.9)	44 (22.1)	15 (18.1)	16 (20.3)	9 (15.8)	3 (10.7)	7 (18.4)	140 (18.1)
**Day**								
Monday	44 (15.2)	19 (9.5)	14 (16.9)	8 (10.1)	4 (7.0)	4 (14.3)	1 (2.6)	94 (12.1)
Tuesday	44 (15.2)	19 (9.5)	16 (19.3)	12 (15.2)	12 (21.1)	2 (7.1)	10 (26.3)	115 (14.8)
Wednesday	40 (13.8)	27 (13.6)	16 (19.3)	11 (13.9)	9 (15.8)	7 (25.0)	9 (23.7)	119 (15.4)
Thursday	40 (13.8)	45 (22.6)	10 (12.0)	11 (13.9)	6 (10.5)	5 (17.9)	9 (23.7)	126 (16.3)
Friday	49 (16.9)	30 (15.1)	12 (14.5)	14 (17.7)	14 (24.6)	4 (14.3)	3 (7.9)	126 (16.3)
Saturday	39 (13.4)	32 (16.1)	10 (12.0)	11 (13.9)	6 (10.5)	2 (7.1)	3 (7.9)	103 (13.3)
Sunday	34 (11.7)	27 (13.6)	5 (6.0)	12 (15.2)	6 (10.5)	4 (14.3)	3 (7.9)	91 (11.8)
**Month**								
August 2014	42 (14.5)	37 (18.6)	14 (16.9)	15 (19.0)	4 (7.0)	-	6 (15.8)	118 (15.2)
September 2014	33 (11.4)	13 (6.5)	7 (8.4)	10 (12.7)	6 (10.5)	1 (3.6)	7 (18.4)	77 (9.9)
October 2014	29 (10.0)	9 (4.5)	6 (7.2)	8 (10.1)	6 (10.5)	-	-	58 (7.5)
November 2014	27 (9.3)	17 (8.5)	2 (2.4)	-	4 (7.0)	2 (7.1)	1 (2.6)	53 (6.8)
December 2014	22 (7.6)	17 (8.5)	5 (6.0)	5 (6.3)	2 (3.5)	1 (3.6)	5 (13.2)	57 (7.4)
January 2015	16 (5.5)	10 (5.0)	4 (4.8)	6 (7.6)	2 (3.5)	10 (35.7)	2 (5.3)	50 (6.5)
February 2015	21 (7.2)	19 (9.5)	3 (3.6)	3 (3.8)	3 (5.3)	3 (10.7)	2 (5.3)	54 (7.0)
March 2015	18 (6.2)	16 (8.0)	6 (7.2)	4 (5.1)	4 (7.0)	2 (7.1)	1 (2.6)	51 (6.6)
April 2015	14 (4.8)	14 (7.0)	9 (10.8)	6 (7.6)	4 (7.0)	3 (10.7)	5 (13.2)	55 (7.1)
May 2015	21 (7.2)	17 (8.5)	8 (9.6)	5 (6.3)	4 (7.0)	2 (7.1)	2 (5.3)	59 (7.6)
June 2015	29 (10.0)	15 (7.5)	8 (9.6)	7 (8.9)	11 (19.3)	2 (7.1)	3 (7.9)	75 (9.7)
July 2015	18 (6.2)	15 (7.5)	11 (13.3)	10 (12.7)	7 (12.3)	2 (7.1)	4 (10.5)	67 (8.7)

Compared with the average distribution across all other methods, self-poisoning (by medication)—the fourth most common method of self-harm—was significantly more likely to occur between 12:00 AM and 3:59 AM (χ^2^ [5, *N* = 774] = 15.4, *p* = 0.009), whereas self-battery was significantly more likely to occur between 8:00 AM and 11:59 AM (χ^2^ [5, *N* = 774] = 20.8, *p* ≤ 0.001). Cutting (χ^2^ [5, *N* = 774] = 5.6, *p* = 0.341) and attempted hanging (χ^2^ [5, *N* = 774] = 6.0, *p* = 0.300) did not vary by time of occurrence.

### Methods of self-harm by day of the week and month of occurrence

There were no significant differences in the method(s) used to self-harm by day of the week (χ^2^ [36, *N* = 774] = 47.2, *p* = 0.100) or in the three most common methods of self-harm, according to month of occurrence (χ^2^ [22, *N* = 572] = 26.1, *p* = 0.246). Table 3 displays the variations in the method(s) used to self-harm across the Australian asylum seeker population by day of the week and month of the year.

## Discussion

In this exploratory study, we examined the distribution of self-harm episodes, and method(s) used to self-harm in the Australian asylum seeker population according to the 24-hour cycle, day of the week, and month of the year over a 12-month period. We observed diurnal and monthly variations in self-harm episodes across the Australian asylum seeker population, including by processing arrangements. Specifically, the four-hourly pattern for community-based asylum seekers was such that self-harm most commonly occurred between 12:00 AM and 3:59 AM, for asylum seekers on Manus Island, between 4:00 PM and 7:59 PM, and for asylum seekers in onshore immigration detention, between 8:00 PM and 11:59 PM. Two of the most common methods of self-harm—self-poisoning (by medication) and self-battery—were also found to vary according to time of the day, occurring most frequently between 12:00 AM and 3:59 AM, and 8:00 AM and 11:59 AM, respectively. Significant monthly variations in self-harm were found in Nauru (in October 2014), in Manus Island (in January 2015), and in onshore detention (in August 2014). Drawing out the features of the various detention policies, practices, and conditions that may have been associated with the observed temporal variations in self-harm in the Australian asylum seeker population may guide future research and self-harm monitoring practices. As these may in turn help to inform self-harm prevention strategies, including at the public policy level, they are important to highlight and are therefore considered in more detail below.

The largest proportion of self-harm incidents across the Australian asylum seeker population occurred between 12:00 PM and 3:59 PM, whilst the trough in self-harm incidents occurred between 4:00 AM to 7:59 AM. Nearly half (46%) of all self-harm incidents occurred between 12:00 PM and 7:59 PM. Whilst the trough in self-harm during the very early hours of the morning is consistent with previous research regarding hospital-treated self-harm presentations [20], the substantial proportion occurring across the entire asylum seeker population during business hours and in the early evening is not. Previous research regarding the timing of self-harm in hospital-treated episodes has found that the peak in self-harm most commonly occurred between 10 PM and midnight [21]. The diurnal pattern observed across the asylum seeker population as a whole is therefore more comparable with recent research from the prison population, which found that just over half (52%) of all self-harm incidents occurred between 2 PM and 8 PM [22]. Detention-specific factors such as the environment, issues related to accommodation, and legal concerns, procedure-related factors such as changes in cells or changes in security level, relationship concerns regarding family and staff, as well as mental and physical health concerns were cited as the most common contributing factors to self-harm occurring in the prison population during these hours of the day [22]. Similar precipitating factors for self-harm amongst asylum seekers in the onshore detention population have, in fact, also been previously identified, with detention-related conditions, procedure-related factors, such as claim processing times, as well as separation from family found to be the most common precipitants [23]. Whilst the present study was not able to measure precipitants for self-harm, it is plausible that similar factors also contributed to self-harm episodes in the present study.

The four-hourly pattern of self-harm according to processing arrangements revealed some further differentiation in diurnal patterns. For community-based asylum seekers, self-harm was significantly more likely to occur between 12:00 AM and 3:59 AM, compared with the average distribution across all other arrangements, which is more consistent with late evening to early morning patterns found in hospital-treated self-harm presentations [20]. Importantly, the early morning peak in self-harm found in community-based asylum seekers highlights the disrupted sleep-wake cycles of this population. Whilst this is likely a consequence of mental health conditions such as depression and/or anxiety, precipitated by the circumstances under which community-based asylum seekers are living—outlined further below—the disrupted sleep-wake cycles are potentially more evident in community-based asylum seekers, as their evening routines are less able to be controlled than in other populations. It is important to note that community-based asylum seekers have less contact with DIBP and other contracted case workers than asylum seekers in closed detention arrangements. It is plausible, therefore, that the early morning peak in self-harm observed amongst community-based asylum seekers is partly an artefact of the reporting and supervisory practices associated with this particular population.

For asylum seekers on Manus Island, self-harm most commonly occurred between 4 PM and 7:59 PM, which is more comparable with patterns found in recent prison research [22], as highlighted above. As services and staffing levels on Manus Island have previously been identified as being inadequate [24], it is plausible that the time of day in which access to physical, mental, and other support services becomes limited corresponds to an increase in self-harm for asylum seekers held there. Furthermore, as threats to asylum seekers’ safety, including acts of violence and intimidation by locals and security personnel, have also been reported as occurring on multiple occasions on Manus Island [25], it is also possible that as the evening approaches, asylum seekers’ distress and fear may intensify, thereby triggering acts of self-harm.

For asylum seekers in onshore detention, self-harm was significantly more likely to occur between 8 PM and 11:59 PM, compared with the average distribution across all other processing arrangements. Our own research has found that rates of self-harm among adult asylum seekers in onshore detention during the same study period—at 257 per 1,000 detained asylum seekers—were 214 times higher than hospital-treated rates of self-harm in the general Australian community [5]. Furthermore, our more recent study [26] has observed that self-harm episodes occur most frequently in types of onshore detention where populations are mixed (such as Immigration Transit Accommodation [ITA] and Alternative Places of Detention [APODs]). These types of detention house both single adult males and females, as well as families, for short-, medium-, and long-term stays. Transfers between and within other facilities reportedly occur very frequently in such detention arrangements—including between onshore and offshore detention—often with little notice, resulting in family separation [26]. Such separations are known to occur between parents and children, couples, as well as extended family members. There are a range of possible explanations worth considering for the observed pattern of self-harm: the state of the centres at this time of the evening, fear of transfer during the night, and lower levels of supervision. Furthermore, as both ITAs and APODs were used for housing families with children, it is likely that attempting to carry out parenting tasks in the late evening in such environments, with little autonomy or flexibility, was experienced as particularly challenging for both detained parents and children [26]. Indeed, research has found that held detention environments, including both APODs and ITAs, frequently contributed to feelings of parental disempowerment, with parents often stripped of control over their everyday routines, including organising mealtimes in the evenings to suit children’s bedtimes or easy access to bottles and nappies, as needed, including late at night [27–28]. Of relevance to the present study is that previous research has found that parental disempowerment creates ongoing feelings of distress and helplessness, leading to a deterioration in mental health [29], which in turn may precipitate acts of self-harm.

Some diurnal variations in the most common methods of self-harm used across the Australian asylum seeker population were also found in the present study. The significant increase in self-harm involving self-poisoning (by medication) between 12:00 AM and 3:59 AM, compared with all other methods, is consistent with previous research that has reported an early morning peak in self-poisoning [30]. This finding points to the disruptive sleep-wake cycles of asylum seekers self-harming during these times, which is likely a consequence of mental health conditions, such as depression and/or anxiety, as previously mentioned. As self-harm incidents between 12:00 AM and 3:59 AM were significantly more likely to occur amongst community-based asylum seekers, it is conceivable that the increase in self-poisoning by medication in this population at this time was a consequence of lower levels of supervision, as well as the greater access that community-based asylum might have to medications, compared with those in held detention.

Whilst not significant, the diurnal patterns observed in self-harm episodes involving cutting and attempted hanging—two of the four most common methods of self-harm found in the present study—are important to comment on. Nearly one in three of all self-harm incidents involving cutting occurred between 12:00 PM and 3:59 PM, whilst over a quarter of all self-harm incidents involving hanging occurred between 4:00 PM and 7:59 PM. These findings likely speak to the availability of means and likelihood of detection. The timing of self-harm incidents involving attempted hanging in the present study is also consistent with previous research that found that individuals with higher suicidal intent were more likely to present from 4 PM until the late evening [20]. It is acknowledged that the present study cannot comment directly on suicidal intent. Further research and enhanced self-harm surveillance processes are needed in order to gain a greater awareness of the characteristics associated with self-harm episodes amongst asylum seekers.

The findings of the present study highlight a monthly variation in the distribution of self-harm across the Australian asylum seeker population during the 12-month study period, according to some processing arrangements. Self-harm was significantly more likely to occur in August 2014 amongst asylum seekers in onshore detention, in October 2014 on Nauru, and in January 2015 on Manus Island. There are a number of possible explanations for the observed monthly variation in the distribution of self-harm across the Australian asylum seeker population during the study period. The possible reintroduction of 3-year Temporary Protection Visas (TPVs), instead of permanent protection visas (PPVs), and the conditions associated with such visas were debated in parliament throughout 2014 [31]. As TPVs have previously been found to cause considerable suffering for refugees due to their being placed in a state of legal uncertainty, as well as ongoing concerns about being sent back to a country where they fear persecution [32], it is highly likely that the potential re-introduction of TPVs was experienced as an additional stressor for onshore asylum seekers at this time. For various reasons, this may have been more intensely experienced during August 2014. During this time frame, some 30,000 asylum seekers who had been impacted by a processing freeze on visas (implemented by the government in response to the Senate previously disallowing the reintroduction of TPVs in December 2013) were also waiting to see if the newly formed Senate in July–early August might lift the bar on the processing of their protection visas [33]. This processing freeze reportedly had a significant, detrimental impact on asylum seekers’ mental health during this time [29], and heightened concerns regarding whether or not it would be lifted may have contributed to the observed pattern of self-harm at this time.

For asylum seekers detained on Nauru, a number of events occurred in late September–October 2014, which likely contributed to the spike in self-harm incidents during this time. Throughout this period, there was continuing uncertainty about asylum seekers’ future that may have been heightened at this time: any remaining hope about applying for an Australian visa was closed by an announcement by the Minister for Immigration in September 2014; also at this time, the Minister indicated that asylum seekers in Nauru may be resettled in Cambodia [34–37].

There are also a range of possible gender-specific stressors that may have contributed to the increases in self-harm at this time. A number of reports and inquiries into conditions in immigration detention during this time have highlighted, for example, that sexual assaults and violence against both women and children were commonplace, and likely contributing factors for acts of self-harm [7,29, 38–39]. Indeed, several allegations of sexual and physical abuse against women and children detained on Nauru were made in mid-September to early October 2014, prompting the announcement of a government investigation on 3 October, which later became the Moss review [39]. Interviews for the Moss review, with both asylum seekers and staff and contractors, took place in Nauru during 25 October–1 November and 12–19 November 2014 [39], which may have further heightened feelings of distress amongst asylum seekers at this time. Acts of sexual and physical violence against male asylum seekers, as previously mentioned, may also have contributed to the pattern of self-harm observed in the present study during these particular times. For males, particularly those detained on Manus Island, announcements regarding the first refugee status determination decisions and associated transfers to locations without adequate settlement services and protection from locals [40] may also have been experienced as an additional stressor at this time.

For asylum seekers detained on Manus Island, unrest reported to have occurred throughout January 2015 [41–42] was likely to have contributed to increased distress and self-harm at this time. Reports from human rights organisations as well as the media indicated that asylum seekers who had been determined to be refugees were informed they were to be transferred to insecure accommodation (in Lorengau, the major town in Manus Province) on 22 January, where they feared they would be vulnerable to further attacks by locals [42]. Unrest and conflict involving up to 300 asylum seekers were subsequently reported as occurring over a number of days, with numbers on a hunger strike eventually reaching over 500 and continuing for over 10 days [42].

Future research should examine these temporal trends in further detail in order to inform self-harm prevention strategies, including those at the policy level. Such research should also more directly investigate any potential links with policies, practices, and conditions. Ongoing self-harm monitoring practices in line with WHO guidelines [11] should also be established to more closely track and identify temporal (and other) trends to protect the health and safety of asylum seekers.

It is important to mention something briefly here about understandings of self-harm. Self-harm is a behaviour, understood as a way of coping with or expressing overwhelming distress [43]. As outlined clearly by clinical experts and allied and other health professionals in Australian government inquiries into self-harm among asylum seekers, self-harm is generally observed to occur as a consequence or way of managing genuine psychological distress and not as a form of manipulative behaviour [7,15].

### Strengths and limitations

Our study had a number of strengths. First, these are the first published data examining the variation in self-harm incidents and method(s) of self-harm used across the Australian asylum seeker population, by hour, day of the week, and month of occurrence, according to WHO self-harm reporting guidelines [11]. Second, we were able to access—via the *Freedom of Information Act*—all self-harm incidents recorded as occurring across the Australian asylum seeker population during the 12-month study period. Third, our sample was large and enabled us to not only examine the temporal patterns in self-harm, as well as method(s) used to self-harm across the entire Australian asylum seeker population, but also the temporal dimensions of self-harm according to each of the five main processing arrangements (i.e., community-based asylum arrangements, community detention, onshore detention, offshore detention [Nauru], and offshore detention [Manus Island]).

Our study also had some limitations. The number of unreported self-harm incidents during the study period is unknown. It is likely, therefore, that the self-harm incidents reported here are an underestimate of the real number of self-harm incidents occurring across the Australian asylum seeker population. Furthermore, some self-harm incidents, particularly those requiring little or no medical treatment, may not have come to the attention of staff immediately. Under these circumstances, it is likely that the time of occurrence logged by staff on the self-harm incident reports would have been an estimation. There was also the potential risk of making a type 1 error, given the number of exploratory comparisons we undertook, although we minimised this risk by lowering our significance threshold from 0.05 to 0.01 [19]. We were also limited in our ability to investigate certain potentially important characteristics associated with self-harm, as the DIBP did not routinely require such data to be collected. Information relating to gender, for example, could only be extracted from just over two thirds of the self-harm incident reports, meaning that some processing arrangements had higher proportions of missing gender data than other processing arrangements. This restricted our ability to reliably investigate any further interactions between gender and temporal dimensions. Information relating to precipitating factors for self-harm was also only able to be extracted from less than 10% of all self-harm incident reports. Furthermore, whilst mental health among asylum seekers in Australian immigration detention has previously been found to deteriorate with length of detention [44], information regarding time spent in detention was not recorded on the incident reports. Identifying information such as country of origin, age, and date of birth would have been redacted prior to the incident reports being released under FOI. In addition to this, information regarding psychiatric history and suicidal intent—which have previously been found to be associated with self-harm in the general population [45]—was not able to be extracted from the self-harm incident reports. The data analysed also did not include instances of suicide, as deaths are not recorded in this category of incident reports. As noted in the Methods section, there were 3 deaths by suicide during the study period [16]. Whilst these deaths may speak to neglect and/or other forms of structural abuse, and are clearly of concern, the inclusion of these reported suicides would not be likely to impact patterns in the self-harm data, and the low number of deaths by suicide preclude analysis of temporal patterns. Lastly, the low numbers of self-harm observations meant that we were unable to detect statistically significant differences in self-harm among asylum seekers in community detention by month of occurrence. This meant that we were not able to ascertain whether self-harm among asylum seekers in community detention varied by month of the year, or make meaningful comparisons regarding monthly variations in self-harm across all processing arrangements.

### Conclusions

Self-harm in the Australian asylum seeker population was found to vary according to time of day and month of the year, including by processing arrangements. Given the harms observed across the Australian asylum seeker population—which we have previously asserted constitute a public health crisis [12]—the current findings should serve as a basis for more detailed investigation into temporal variations in self-harm among asylum seekers, which may in turn lead to effective self-harm prevention strategies.

## Supporting information

S1 STROBE Checklist STROBE, Strengthening the Reporting of Observational Studies in Epidemiology. (DOCX)

S1 Table Monthly population figures, self-harm episode rates, with 95% CIs, between 1 August 2014 and 31 July 2015, for the Australian asylum seeker population. CI, confidence interval
(DOCX)

S2 Table Monthly population figures, self-harm episode rates, with 95% CIs, between 1 August 2014 and 31 July 2015, for asylum seekers in onshore detention, Nauru and Manus Island. CI, confidence interval
(DOCX)

## Abbreviations

APODs: Alternative Places of Detention
CI: confidence interval
DIBP: Department of Immigration and Border Protection
FOI: Freedom of Information
ITA: Immigration Transit Accommodation
PNG: Papua New Guinea
PPV: permanent protection visa
PTSD: post-traumatic stress disorder
STROBE: Strengthening the Reporting of Observational Studies in Epidemiology
TPV: Temporary Protection Visa
WHO: World Health Organization
