# Supplementary material for: Temporal variations in the distribution of self-harm episodes and methods across the Australian asylum seeker population: An observational study
Source: PLoS Med. 2020 Aug 6;17(8):e1003235. doi: 10.1371/journal.pmed.1003235 (PMC7410206; doi:10.1371/journal.pmed.1003235)
Supplement: S1 Table — CI, confidence interval (DOCX) [file pmed.1003235.s002.docx]

**S1 Table. Monthly population figures, self-harm episode rates, with 95% Confidence Intervals, between 1 August 2014 and 31 July 2015, for the Australian asylum seeker population**

| Month of the year | Total Australian asylum seeker population | Self-harm  (*N* = 949) | Self-harm episode rate per 1,000 | 95% Confidence Intervals |
| --- | --- | --- | --- | --- |
| AUG 2014 | 29062 | 148 | 5.0 | 1.6-11.6 |
| SEP 2014 | 28961 | 93 | 3.2 | 1.0-8.7 |
| OCT 2014 | 28979 | 79 | 2.7 | 0.6-7.2 |
| NOV 2014 | 28960 | 69 | 2.3 | 0.6-7.2 |
| DEC 2014 | 29037 | 69 | 2.3 | 0.6-7.2 |
| JAN 2015 | 29001 | 62 | 2.1 | 0.6-7.2 |
| FEB 2015 | 29038 | 62 | 2.1 | 0.6-7.2 |
| MAR 2015 | 29014 | 65 | 2.2 | 0.6-7.2 |
| APR 2015 | 29010 | 70 | 2.4 | 0.6-7.2 |
| MAY 2015 | 29003 | 69 | 2.3 | 0.6-7.2 |
| JUN 2015 | 28954 | 86 | 2.9 | 0.6-7.2 |
| JUL 2015 | 28913 | 77 | 2.6 | 0.6-7.2 |
