# Supplementary material for: Temporal variations in the distribution of self-harm episodes and methods across the Australian asylum seeker population: An observational study
Source: PLoS Med. 2020 Aug 6;17(8):e1003235. doi: 10.1371/journal.pmed.1003235 (PMC7410206; doi:10.1371/journal.pmed.1003235)
Supplement: S2 Table — CI, confidence interval (DOCX) [file pmed.1003235.s003.docx]

**S2 Table. Monthly population figures, self-harm episode rates, with 95% Confidence Intervals, between 1 August 2014 and 31 July 2015, for asylum seekers in onshore detention, Nauru and Manus Island.**

| Month of the year | Onshore detention population | Self-harm episodes  (*N* = 560) | Episode rate of self-harm per 1,000 | 95% CI | Nauru  population | Self-harm episodes  (*N* = 188) | Episode rate of self-harm per 1,000 | 95% CI | Manus  Island population | Self-harm episodes  (*N* = 55) | Episode rate of self-harm per 1,000 | 95% CI |
| --- | --- | --- | --- | --- | --- | --- | --- | --- | --- | --- | --- | --- |
| AUG 2014 | 2793 | 109 | 39.0 | 27.7-53.3 | 1011 | 12 | 11.8 | 6.2-19.6 | 1084 | 4 | 3.6 | 1.0-8.7 |
| SEP 2014 | 2711 | 57 | 21.0 | 12.9-32.1 | 954 | 19 | 19.9 | 12.2-29.6 | 1060 | 1 | 0.9 | 0.02-3.0 |
| OCT 2014 | 2525 | 30 | 12.0 | 6.2-20.9 | 928 | 29 | 31.2 | 21.8-44.0 | 1056 | 1 | 0.9 | 0.02-3.0 |
| NOV 2014 | 2620 | 41 | 15.6 | 9.1-24.7 | 841 | 12 | 14.2 | 8.3-23.4 | 1044 | 7 | 6.7 | 2.8-13.0 |
| DEC 2014 | 2337 | 40 | 17.1 | 10.6-27.2 | 760 | 12 | 15.7 | 9.1-24.7 | 1035 | 6 | 5.7 | 2.2-11.6 |
| JAN 2015 | 2087 | 25 | 12.0 | 6.2-20.9 | 683 | 17 | 24.8 | 16.1-35.7 | 1023 | 10 | 9.7 | 4.7-17.0 |
| FEB 2015 | 1861 | 37 | 20.0 | 12.2-30.8 | 635 | 15 | 23.6 | 15.3-34.5 | 1004 | 3 | 2.9 | 0.6-7.2 |
| MAR 2015 | 1724 | 33 | 19.1 | 12.2-29.6 | 615 | 17 | 27.6 | 18.6-39.2 | 989 | 6 | 6.0 | 2.2-13.0 |
| APR 2015 | 1787 | 48 | 27.0 | 17.7-39.2 | 582 | 15 | 25.7 | 16.9-36-9 | 971 | 2 | 2.0 | 0.2-7.2 |
| MAY 2015 | 1888 | 39 | 20.6 | 12.9-30.8 | 553 | 11 | 19.8 | 12.2-29.6 | 943 | 7 | 7.4 | 3.4-14.4 |
| JUN 2015 | 1886 | 55 | 29.1 | 20.2-41.6 | 567 | 13 | 23.0 | 14.5-34.5 | 945 | 4 | 4.2 | 1.6-10.2 |
| JUL 2015 | 1897 | 46 | 24.2 | 16.1-35.7 | 550 | 16 | 29.0 | 19.4-41.6 | 942 | 4 | 4.2 | 1.6-10.2 |
